# YAP condensates are highly organized hubs

**DOI:** 10.1016/j.isci.2025.114528

**Published:** 2025-12-29

**Authors:** Siyuan Hao, Ye Jin Lee, Nadav Benhamou Goldfajn, Eduardo Flores, Jindayi Liang, Hannah Fuehrer, Justin Demmerle, Jennifer Lippincott-Schwartz, Zhe Liu, Shahar Sukenik, Danfeng Cai

## Main text

(iScience *27*, 109927; June 21, 2024)

Due to errors introduced during the drafting of the manuscript by Dr. Cai, an image duplication was inadvertently introduced into Figure S2. The data in panel O pertaining to TEAD1 concentration 8 μM were duplicated, and the data for TEAD1 concentration 4 μM were excluded. The authors apologize for this oversight. The figure has been corrected in the published supplementary file.

**Figure:**
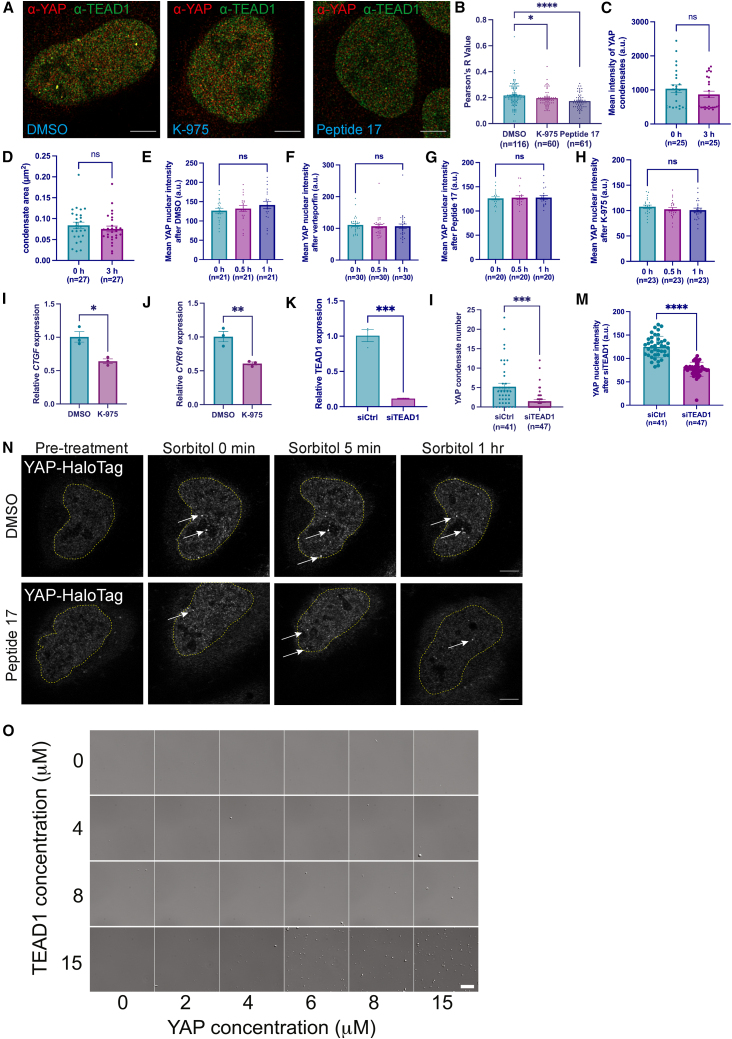
Figure S2. TEAD1 transcription factor stabilizes YAP condensate, related to Figure 2 (corrected)

